# Supplementing Silicoglycidol for the reduction of aflatoxin M_1_ in milk and biomarkers of liver dysfunction in dairy cows

**DOI:** 10.3168/jdsc.2023-0538

**Published:** 2024-03-29

**Authors:** E.H. Branstad-Spates, C.S. McCarthy, B.C. Dooley, L.E. King, E.L. Bowers, A. Tesouro, J. Borrell, D. Díez, G.E. Rottinghaus, L.H. Baumgard

**Affiliations:** 1 Department of Animal Science, Iowa State University, Ames, IA 50011; 2 Department of Agriculture and Biosystems Engineering, Iowa State University, Ames, IA 50011; 3 Biovet, S.A., Tarragona, Spain, 43003; 4 Veterinary Medical Diagnostic Laboratory, University of Missouri, Columbia, MO 65211

## Abstract

Aflatoxin M_1_ (AFM_1_) is a pathogenic metabolite transferred from feed into milk from aflatoxin (AF) B_1_, B_2_, G_1_, and G_2_; thus, it poses a human health risk. Therefore, effective mitigation strategies are needed to reduce animal and human exposure to AF. Study objectives were to evaluate a dietary adsorbent (Silicoglycidol, ATX) as a sequestering agent in AF-contaminated feed and to broadly examine how AF affects liver function and the immune system. Primiparous Holstein cows (n = 12; 279 ± 88 DIM and 675 ± 19 kg BW) were used in a replicated 3 × 3 Latin square design with 21-d periods in which d 1 to 14 were considered adaptation, and data collected on d 15 to 21 were used for analysis. Treatments were (1) control (CON) consisting of a basal diet, (2) AF diet consisting of CON+AF challenge (100 µg of AFB_1_/kg DMI), and (3) AF+ATX supplemented at 0.10% of dietary DMI. Feed intake and milk yield were recorded daily, fecal samples were collected on d 20 of each period, blood and urine samples were collected on d 21 of each period, and milk samples were collected on the last 2 d of each period. Data were analyzed using the MIXED procedure of SAS (SAS Institute Inc.). Milk yield and DMI were unaffected by treatment (26.8 ± 1.3 kg/d and 24.0 ± 0.9 kg/d, respectively). Similarly, neither milk composition nor DMI digestibility were affected by treatment. No AFM_1_ was detected in CON cow milk or urine. Supplementing ATX reduced AFM_1_ in milk (1.57 vs. 1.14 ± 0.1 µg/L for AF and AF+ATX, respectively) and urine (9.9 vs. 5.6 ± 1.1 µg/L for AF and AF+ATX, respectively). Consuming AF did not affect biomarkers of liver health or immune activation including alanine aminotransferase, aspartate aminotransferase, gamma glutamyltransferase, haptoglobin, and IgG. In summary, feeding ATX reduced the absorption and transfer of dietary AF to milk and urine.

## Highlights

• Dietary AF transfers to milk.
• Aflatoxin ingestion did not affect health or production in lactating dairy cows.
• Silicoglycidol supplementation reduced the alimentary tract absorption of AF.

## Graphical Abstract

**Figure:**
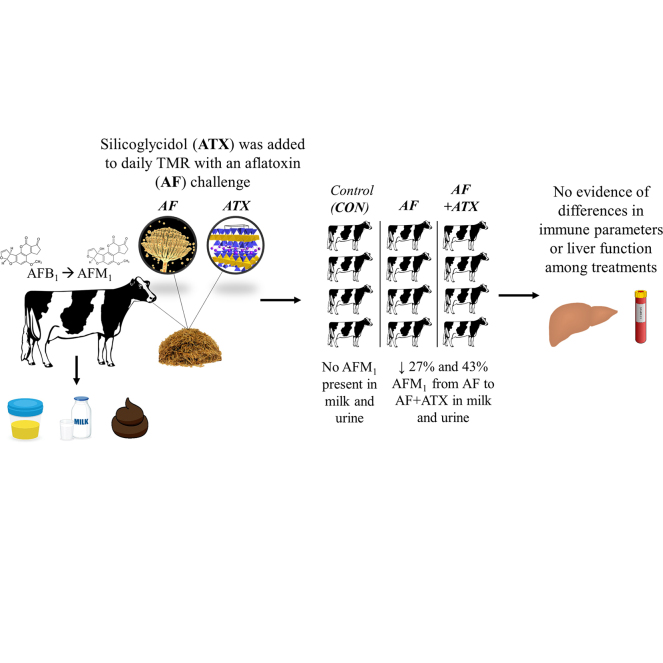
**Summary:** Aflatoxin (AF) is a toxic secondary metabolite produced from filamentous fungi in food and feed commodities. Due to the transfer of aflatoxin M1 (AFM1) from feed into milk, there is an important human risk. Thus, this study evaluated a dietary adsorbent (Silicoglycidol [ATX]) as a sequestering agent in AF-contaminated feedstuffs and how AF affected liver and immune system function. Surprisingly, milk yield, milk composition, dry matter intake, and digestibility were unaffected by AF. Dietary AF was transferred into both urine and milk, and supplementing ATX markedly reduced AFM1 in milk and urine. Consuming AF did not affect biomarkers of liver health or immune activation. In summary, feeding ATX reduced the absorption and transfer of dietary AF to milk and urine.

The USDA and Food and Drug Administration (**FDA**) spend approximately $31.1 million in mitigation strategies for mycotoxins annually (Robens and Cardwell, 2003). Within the mycotoxin umbrella, aflatoxins (**AF**) are of special concern; these are primarily produced by the fungi *Aspergillus flavus* and *Aspergillus parasiticus*, and about 25% of global cereal grains (primarily corn) are AF-contaminated (IARC, 2002; FAO, 2004). When animals consume AFB_1_ (an AF derivative), it is hepatically bio-transformed into AFM_1_ (a secondary metabolite), which is excreted in urine and milk (Fink-Gremmels, 2008). Because AF and its derivatives can be pathogenic and immunosuppressive, human health is a concern, principally with AFM_1_, because it is a more potent toxin compared with its parent molecule (Abrar et al., 2013). To ensure a safe food supply, the FDA has set an action threshold of 20 μg of AF/kg of DM in lactating dairy cow rations and 0.5 μg AF/L of milk (US Food and Drug Administration, 2000).

Feed additives, such as adsorbents, can inactivate or detoxify mycotoxins like AF within the gastrointestinal tract (Huwig et al., 2001). There are a wide variety of potential adsorbents, differing in the physical structure of the constituent molecules, electrical charge, charge distribution, accessible surface area, and pore size (Huwig et al., 2001). Most dairy nutrition studies have focused on aluminosilicates (primarily hydrated sodium calcium aluminosilicates; **HSCAS**), zeolites, bentonites, and activated charcoal. Phillips et al. (1988) demonstrated that HSCAS can absorb 80% of AFB_1_ in vitro, but this efficacy may be dose-dependent (Smith et al., 1994). However, AFB_1_ can be physically resilient to binders, seemingly contributing to the variation in adsorption properties of the aforementioned clays (Vekiru et al., 2007). Furthermore, most adsorbents are effective for AF adsorption but may have little affinity for other toxins such as ochratoxin A (**OTA**), fumonisin (**FUM**), zearalenone (**ZEA**), and deoxynivalenol (**DON**) (Huff et al., 1992; Patterson and Young, 1993).

Silicoglycidol (**ATX**) binds mycotoxins by forming hydrogen bonds between oxygen atoms of silicate (SiO_4_; Biovet, 2021). Low doses of ATX bind more than one mycotoxin in swine (Binder et al., 2017), but to our knowledge, there are no reports of its efficacy in dairy cows. Therefore, the study objectives were to determine the effects of ATX on cow productivity, AFM_1_ concentrations in milk and urine, and broad proxies of liver and immune function during an AF challenge in dairy cows. We postulated that supplementing ATX would reduce intestinal AFB_1_ absorption, thus reducing AFM_1_ secretion in milk and urine, and that this would be coupled with increased production, improved markers of liver health, and a reduced acute phase protein response.

All procedures were approved by the Institutional Animal Care and Use Committee of Iowa State University (Ames, IA). Twelve primiparous Holstein cows (279 ± 88 DIM and 675 ± 19 kg BW; mean ± SD) were used in a replicated 3 × 3 Latin square design with 21-d periods. In each period, cows within a square were randomly assigned to 1 of 3 dietary treatments: (1) control (**CON**), consisting of a basal TMR; (2) AF, consisting of the basal TMR plus the AF challenge; and (3) AF+ATX similar to AF but supplemented with ATX (Alquerfeed Antitox, Biovet S.A., Tarragona, Spain) at 0.10% of dietary DM. Cows were estimated to consume 28 kg of DM of a diet formulated to meet or exceed the predicted requirements (NRC, 2001; Table 1). Therefore, ATX was mixed with an allotted 2.5 kg of TMR at 0.10% of projected DMI. Similarly, the AF challenge was implemented by feeding a daily dose of 100 µg/kg of estimated DMI by mixing in with the allotted 2.5 kg of TMR. The AF was produced as previously described (West et al., 1973). In brief, AF was produced through rice fermentation by *A. parasiticus* NRRL-2999. The resulting rice powder medium was obtained from the University of Missouri Veterinary Medical Diagnostic Laboratory (Columbia, MO) and contained 100 mg/kg AFB_1_/13 kg. The AF-contaminated powder was mixed with ground corn. The profile and the concentration of AF content in the blended powder meal were 103 mg AFB_1_/kg, 3 mg AFG_1_/kg, 35 mg AFB_2_/kg, and 1 mg AFG_2_/kg.

**Table 1:** Ingredient and analyzed chemical composition of the basal diet^1^

Item	Value
Dietary ingredient, % of dietary DM	
Corn silage	23.03
Alfalfa baleage	16.37
Alfalfa hay	4.02
Ground corn	29.64
Dry corn gluten feed	7.89
Soybean meal	4.37
Cottonseed, whole	3.87
Expeller soybean meal	2.38
Molasses	1.79
Protein, vitamin, and mineral premix^2^	6.66
Nutrient analysis, % DM	
CP	17.30
NDF	26.10
ADF	17.73
Starch	27.20
NFC^3^	45.67
Ether extract	4.70
Ash	7.25
Particle size distribution, % retained in each sieve^4^	
>19.0 mm	18.61
19.0–9.0 mm	30.11
8.0–4.0 mm	35.58
<4.0 mm	15.70

1 Values represent an average of composite samples collected throughout the trial (n = 3).

2 Contained soybean meal (SoyPlus, West Central Cooperative, Ralston, IA), calcium carbonate, bloodmeal, sodium bicarbonate, rumen-inert fat (MagnaPalm, Energy Feeds International, Lago Vista, TX), salt, daily micro premix, urea, magnesium oxide, antioxidants/polyphenols (NutriTek Techology, Purina Animal Nutrition, Gray Summit, MO), methionine (Alimet, Novus, Saint Charles, MO), choice white grease, rumen-protected methionine (Smartamine M, Kemin, Des Moines, IA), ionophore (Rumensin, Elanco, Fort Dodge, IA), 2% biotin, and performance minerals (Zinpro 120, Zinpro, Eden Prairie, MN).

3 NFC calculated by the difference of 100 − (% NDF + % CP + % ether extract + % ash).

4 Measured using the Penn State Particle Separator (Nasco, Fort Atkinson, WI).

Cows were housed in a freestall barn equipped with individual feeding gates (Calan Broadbent Feeding System; American Calan, Northwood, NH). Daily care involved milking at 0000, 0900, and 1600 h, and all milk, regardless of treatment, was discarded. Cows were fed a TMR delivered once daily at 0600 h, and an initial aliquot of 2.5 kg was given to the cows with their respective treatment mixed in, with the remainder of feed being offered upon full consumption of the initial aliquot. This approach was to ensure cows received full consumption of respective treatments. Orts were recorded daily 1 h before feed delivery and did not contain AF.

Basal TMR samples were collected on d 20 and 21 of each period and pooled to obtain a composite sample by period. The diet was analyzed for nutrient composition (Cumberland Valley Analytical Services, Waynesboro, PA). The ingredient composition and analyzed nutrient content of the basal TMR are presented in Table 1.

Indigestible neutral detergent fiber (**iNDF**) was used as an internal marker to estimate fecal output to determine apparent total-tract digestibility based on fecal samples collected on d 20 of each period. Dry and ground fecal samples were collected from each cow and iNDF determined as previous described (Huhtanen et al., 1994). Urine samples (n = 1/cow) were collected on d 21 of each period and stored at −20°C before being submitted to the Department of Veterinary Pathology at Iowa State University (Ames, IA) to determine creatinine to estimate urinary output (Leonardi et al., 2003).

Individual milk production was measured and recorded daily, with the last 7 d of each period used to evaluate milk production. Individual milk samples for composition analysis were obtained at 6 consecutive milking shifts on d 20 and 21 of each period. Samples were stored at 4°C with a preservative (Bronopol tablet; D & F Control System, San Ramon, CA) until analysis. Milk samples were analyzed (Dairy Lab Services, Dubuque, IA) and yields of milk components were estimated according to milk weight and collection time.

All blood samples were collected coccygeal venipuncture and acquired on d 21 of each period following. All samples were obtained using K_2_EDTA and K_3_EDTA (BD, Franklin Lakes, NJ) 10- and 3-mL vacuum tubes, respectively. Plasma was harvested following centrifugation at 1,500 × *g* for 15 min at 4°C and subsequently frozen at −20°C until analysis. Samples for complete blood count analysis were stored at 4°C for approximately 12 h before being submitted to the Department of Veterinary Pathology at Iowa State University (Ames, IA). The same laboratory analyzed samples for hepatic enzymes, namely alanine aminotransferase (**ALT**), aspartate aminotransferase (**AST**), and gamma glutamyltransferase (**GGT**). Haptoglobin (**Hp**) and IgG were determined using commercially available kits according to the manufacturers' instructions (Hp, Immunology Consultants Laboratory Inc., Lake Oswego, OR; IgG, Bethyl Laboratories Inc., Montgomery, TX).

For AF quantification, milk samples were analyzed by the University of Missouri Veterinary Diagnostic Laboratory (Columbia, MO). Milk samples were thawed and centrifuged at 1,875 × *g* at 4°C for 15 min and milk fat was discarded. The milk filtrates (10 mL) were then passed through AFLAPREP M immunoaffinity cleanup columns (R-Biopharm Rhone Ltd., Glasgow, Scotland). Columns were washed twice with 10 mL of PBS, and AFM_1_ was then eluted from the columns with 1.5 mL of acetonitrile, followed by 1.5 mL of water. These elution fractions were placed in auto-sample vials and analyzed by HPLC with fluorescence detection. The HPLC system consisted of a Hitachi Model L-7100 pump, a Hitachi Model L-7485 fluorescence detector (365 nm excitation and 440 nm emission), a Hitachi Model L-7200 autosampler, a Hitachi D-7000 data acquisition interface, and Concert Chrom software on a microcomputer. The HPLC column was a 150 × 4.6 mm reversed-phase HyperClone 3-μm C_18_ BDS column (Phenomenex) with a C_18_ Security Guard precolumn (Phenomenex). The mobile phase was acetonitrile:methanol:water (15:15:70), and the flow rate was 1 mL/min. The injection volume was 50 μL for all standards and samples. The detection limit was set at 40 ng/kg of AFM_1_. Urine samples were thawed and centrifuged at 1,875 × *g* at 4°C for 15 min. The urine filtrates (2 mL) were then processed and analyzed as previously described for milk samples.

The first 14 d of each period were considered an adaptation/washout phase to avoid carry-over effects, whereas d 15 through 21 were used for data collection. All milk yield and DMI were condensed to means of the last 7 and 2 d of each period for analyses.

Fat-corrected milk was calculated as described by Tyrrell and Reid (1965). Feed efficiency (**FE**) was calculated as FE = milk yield ÷ DMI. Dry matter digestibility (DIG) was calculated as DIG % = [100 − (100 × iNDF TMR% ÷ iNDF fecal %)]. Urine output (L/d) was calculated using estimations from Whittet (2004), assuming averages of 28 mg/kg of BW. The AF excretion and transfer were calculated based on milk production on the day of collection according to the following equations:

AF excretion (µg/d) = concentration of AF in milk (µg/L) × milk yield (kg/d),

AFtransfer%=(AFexcretion(μg/μgdd)AFintake(μg/μgdd))×100.

Urine AF excretion, urine transfer, and total fluid transfer (milk + urine) were calculated using the following equations:

AF excretion (µg/d) = concentration of AF in urine (µg/L) × urine output (L/d),

AFtransfer%=(AFexcretioninurine(μg/μgdd)AFintake(μg/μgdd))×100,and

AFtransferintotalfluid%=(AFexcretioninurine(μg/μgdd)+milk(μg/μgdd)AFintake(μg/μgdd))×100.

Data were analyzed as replicated 3 × 3 Latin squares using the MIXED procedure of SAS (version 9.4, SAS Institute Inc., Cary, NC). The fixed effects of the model included square, period within square, and treatment. Random-effects included cow within square. All squares were balanced to allow for determination of carryover effects between periods (Ratkowsky et al., 1992). The linear model for these data is written as follows:

y_ijkm_ = μ + τ_m_ + β(τ)_im_ + ρ(τ)_jm_ + α_k_ + ε_ijkm_,

where y_ijkm_ is the observation ijkm, μ represents the overall mean, τ_m_ represents the fixed effect of square m, β(τ)_im_ represents the random effect of cow i within square m, ρ(τ)_jm_ represents the fixed effect of period j within square m, and α_k_ represents the fixed effect of treatment. The error term ε_ijkm_ was assumed to be normally, independently, and identically distributed, with variance $\sigma_{e}^{2}.$ Statistical significance for all treatment effects was declared at *P* ≤ 0.05. Trends are discussed at *P* ≤ 0.10. Data are presented as LSM ± the largest SEM unless stated otherwise.

Neither DMI (24.0 ± 0.9 kg/d) nor milk yield (26.8 ± 1.3 kg/d) were affected by treatment (*P* > 0.19). Milk concentrations of fat, protein, lactose, MUN, and SCC were not significantly affected (*P* > 0.21) between treatments (Table 2). Body weight was unaffected (*P* > 0.34) by treatment, and no treatment differences (*P* = 0.32) were detectable for DM digestibility (62.68 ± 2.60%). Likewise, urine (*P* = 0.24) and fecal output (*P* = 0.39) were not affected across all dietary treatments (57.6 ± 8.5 L/d and 9.3 ± 0.8 kg DM/d, respectively).

**Table 2:** Effect of dietary addition of Silicoglycidol (ATX)^1^ on the performance of dairy cows consuming an aflatoxin (AF)-challenge diet

Parameter	Dietary treatment^2^			SEM^3^	*P*-value^4^
	CON	AF	AF+ATX		
DMI, kg/d	24.61	23.66	23.79	0.92	0.19
Milk yield, kg/d	26.90	26.65	26.88	1.35	0.87
3.5% FCM,^5^ kg/d	33.75	32.87	32.60	1.55	0.37
Fat, %	5.15	5.08	5.12	0.11	0.83
Fat, kg	1.37	1.33	1.32	0.06	0.36
Protein, %	3.81	3.66	3.65	0.11	0.47
Protein, kg	1.02	0.96	0.94	0.07	0.27
Lactose, %	4.88	4.92	4.92	0.03	0.21
Lactose, kg	1.30	1.29	1.28	0.07	0.72
MUN, mg/dL	13.45	13.36	13.53	0.44	0.90
SCC (× 1,000)	131	158	253	102	0.49
DM digestibility, %	59.43	65.12	63.50	2.60	0.23

1 Alquerfeed Antitox (Biovet S.A., Tarragona, Spain).

2 CON = control; AF = aflatoxin challenge; AF+ATX = aflatoxin challenge + Silicoglycidol.

3 Greatest standard error of treatment mean is shown.

4 Main effect of treatment.

5 3.5% FCM = [milk fat (kg) × 16.216] + [milk yield (kg) × 0.4324].

No AFM_1_ was detected in milk or urine from cows consuming the CON diet, indicating that the washout period was sufficient for effective AF clearance, comparable to other studies (Sulzberger et al., 2017). The AF-fed cows had 1.6 ± 0.1 µg of AFM_1_/L of milk and feeding ATX reduced (27%) milk AFM_1_ concentrations (*P* ≤ 0.01; Table 3). Total milk output of AFM_1_ markedly decreased in AF-ATX fed cows (41.3 vs. 29.7 µg/d; *P* < 0.01; Table 3). Urine AFM_1_ was 9.9 ± 1.1 µg/L for AF-fed cows and this was reduced (43%) by feeding ATX (*P* ≤ 0.01, Table 3). Total urine AFM_1_ excretion was decreased (*P* = 0.03; Table 3) by 368 µg/d in ATX-fed cows. Total transfer of AFM_1_ (milk + urine) decreased in ATX compared with AF-fed cows (10.81% vs. 25.99%; *P* = 0.02; Table 3). Complete blood cell counts were not affected across all treatments (*P* ≥ 0.20; Table 3). No treatment differences were detected on circulating ALT, AST, GGT, Hp, or IgG (*P* ≥ 0.19; Table 3).

**Table 3:** Effect of dietary addition of Silicoglycidol (ATX)^1^ on AFM_1_ content in milk, urine, blood concentration of hepatic enzymes, white blood cell counts, and immune biomarkers from dairy cows consuming an aflatoxin (AF)-challenge diet

Parameter	Dietary treatment^2^			SEM^3^	*P*-value^4^
	CON	AF	AF+ATX		
Intake of AF, μg/d	—5	2,500	2,500	—	—
Intake of ATX, g/d	—	0.0	28.3	—	—
Milk data					
AFM_1_, μg/L	—	1.57	1.14	0.10	<0.01
Transfer, %	—	1.65	1.19	0.11	<0.01
Excretion, μg/d	—	41.33	29.69	2.64	<0.01
Urine data					
AFM_1_, μg/L	—	9.91	5.64	1.09	<0.01
Transfer, %	—	24.34	9.58	5.57	0.03
Excretion, μg/d	—	608.44	239.47	139.30	0.02
Total excretion, μg/d	—	649.77	270.23	139.23	0.02
Total transfer, %	—	25.99	10.81	6.00	0.02
Enzyme,^6^ IU/L					
ALT	61.5	61.0	60.8	3.61	0.93
AST	65.8	74.9	77.1	5.40	0.19
GGT	79.3	76.6	76.3	7.12	0.36
Immune biomarker					
Hp, μg/mL	1.33	0.85	1.19	0.38	0.55
IgG, μg/mL	155.7	159.6	158.7	6.58	0.70
Cell type, × 10^3^/μL					
White blood cells	9.23	9.59	9.18	1.22	0.76
Neutrophils	3.75	3.83	3.70	0.52	0.97
Lymphocytes	4.37	4.63	4.53	0.88	0.35
Monocytes	0.52	0.53	0.46	0.06	0.59
Eosinophils	0.45	0.40	0.32	0.05	0.20
Basophils	0.10	0.10	0.11	0.02	0.74

1 Alquerfeed Antitox (Biovet S.A., Tarragona, Spain).

2 CON = basal TMR; AF = basal TMR + 100 μg of AFB_1_/kg DMI; AF+ATX = basal TMR + 28.33 g of Silicoglycidol + 100 μg of AFB_1_/kg DMI.

3 Greatest standard error of treatment mean is shown.

4 Main effect of treatment.

5 Not detected.

6 ALT = alanine aminotransferase; AST = aspartate aminotransferase; GGT = gamma glutamyltransferase.

Surprisingly, feeding AF did not affect DMI, milk yield, and milk components. While unexpected, these observations agree with other reports in which a similar amount of AF did not affect DMI, FE, milk yield, and composition (Xiong et al., 2015; Maki et al., 2016a,b; Ogunade et al., 2016). Although the production responses and circulating biomarkers (discussed below) suggest that dairy cows are relatively resilient to AF, feeding a similar quantity of AF-contaminated feed did reduce performance in other dairy studies (Queiroz et al., 2012; Sulzberger et al., 2017; Pate et al., 2018). Notably, for most studies, the experimental contamination levels with AF are similar (∼100 µg AF/kg) and well above the FDA threshold for complete feed (20 μg AF/kg). Unfortunately, the reasons for inconsistent responses among studies are poorly understood but potentially include varying experimental designs and methodologies, such as length of the challenge, timing of sampling, and delivery route of the challenge.

Clay-based feed adsorbents efficiently decrease milk AFM_1_ concentration. For example, calcium montmorillonite clay reduced milk AFM_1_ concentrations by more than 48% using primiparous cows, comparable to this study (Maki et al., 2016a,b). Calcium montmorillonite clay is a highly specific and effective AFB_1_ sorbent; the 2:1 layered calcium montmorillonite structure can tightly bind to AFB_1_ in the interlayer surfaces, resulting in reduced unbound toxin in the gastrointestinal tract (Wang et al., 2017). Xiong et al. (2015) reported a 16.0% reduction of AFM_1_ concentration with the addition of a similar adsorbent (sodium montmorillonite) at 0.25% of dietary DM when cows consumed contaminated diets with 20 to 40 μg AFB_1_/kg. The results of the study herein agree with the aforementioned study, as milk AFB_1_ was markedly reduced. Collectively, adsorbents are an effective way to ameliorate (but not eliminate) AF milk contamination in dairy cows.

Similar to milk, urine AF concentration was markedly decreased by ATX, and this agrees with others (Sulzberger et al., 2017; Rodrigues et al., 2019). Very few experiments report the concentration of AFM_1_ in bovine urine, and even fewer studies report total urinary AFM_1_ excretion. From our results, it is evident that most (94%) AFM_1_ is excreted via urine; in contrast, mammary uptake and secretion is a minor route of AF removal, and this agrees with a previous report (Rodrigues et al., 2019). The factors regulating the relative uptake of AFM_1_ by the kidney and mammary gland and how they are affected by AF and mycotoxin binders are unknown (Rodrigues et al., 2019).

Mycotoxins can have several effects on immunity variables and blood parameters (Xiong et al., 2015; Ogunade et al., 2016; Pate et al., 2018). For example, Ogunade et al. (2016) reported that feeding AF reduced red blood cell counts and hemoglobin concentration, and increased acid-soluble proteins, but did not affect total white blood cells, neutrophils, eosinophils, lymphocytes, and basophils. Herein, there were no differences in circulating leukocyte concentrations, IgG, or Hp. Similarly, Xiong et al. (2015) reported no differences in IgG concentration when dairy cows consumed 20 to 40 µg/kg DM with a 7-d AF challenge. Increased concentrations of liver enzymes can indicate acute or chronic liver disease (Weemhoff et al., 2017; Pate et al., 2018), so we further investigated the hepatotoxic effects of AF by measuring circulating ALT, AST, and GGT. Contrary to expectations, AST, ALT, and GGT were unaffected by treatment, and the lack of differences in both liver function and immune markers, coupled with no adverse effects on productivity, suggests the AF challenge was not pathogenic (at least at the dose we provided).

Haptoglobin is an acute-phase protein that increases during inflammation and is an indicator of the innate immune response (Bertoni et al., 2008; Queiroz et al., 2012); thus, we expected it to be elevated during an AF challenge. Contrary to this, we did not observe differences in Hp concentration even when feeding AF alone. Interestingly, Ryan et al. (2017) observed a 2.27-fold downregulation in Hp gene expression when comparing an AF challenge diet to their AF+ATX treatment. Gaining a better understanding of how or if AF affects liver health and the immune system has obvious implications for pragmatic dairy nutrition and health.

Two unique aspects of the current study are worth highlighting: to the best of our knowledge, this is the first documented report using ATX to mitigate AF in dairy cows. Second, our results indicate that this compound effectively reduces AF absorption when fed at inclusion rates 4 to 10 times lower than other clay-based adsorbents. Previous studies supplemented 100 to 500 g/cow per d of clay adsorbent (Kutz et al., 2009; Sulzberger et al., 2017), whereas our supplementation rate was 28 g/cow per d. This is related to the ATX forming hydrogen bonds between the oxygen atoms of silicate (SiO_4_) and the mycotoxin itself because of the optimized structure of the molecule achieved thanks to the activation patented process (Biovet, 2021). Further research is warranted to determine the potential of ATX to bind other mycotoxins, including DON, FUM, OTA, and ZEA.
